# Supplementary material for: Characterization of Key Odorants in Scallion Pancake and Investigation on Their Changes during Storage
Source: Molecules. 2021 Dec 17;26(24):7647. doi: 10.3390/molecules26247647 (PMC8704002; doi:10.3390/molecules26247647)
Supplement: Supplementary file 1 [file molecules-26-07647-s001.zip › molecules-1482319-supplementary.pdf]

Syntheses of (Z/E)-1-Propenyl propyl trisulfide and (Z/E)-3,6-Diethyl-1,2,4,5-tetrathiane.

$(NH_4)_2S$ (235 mL, 20% in H_2O) was added into the mixture of re-distillated propanal (19.45 g) and THF (125 mL); the mixture obtained was stirred for 4h at room temperature. After reaction, diethyl ether (100 mL) was added, and then organic phase was separated from aqueous phase. Another portion diethyl ether (100 mL) was used for extracting aqueous phase. The combined organic layer was washed with deionized water (100 mL×3), and excess anhydrous sodium sulfate was used for drying. The filtered solution was placed in a rotary evaporator to remove extra solvent until about 20 g intermediate consisting mainly of 2,4,6-triethyl-1,3,5-dithiazinane was obtained. To acquire the target product, 150 mL sodium salt solution (consisting of 14.1 g $NaBrO_3$ and 9.6 g NaBr) was added into a mixture of 100 mL THF and the intermediate obtained; the pH value of the mixture above was adjusted to 1 with 1N HCl drop by drop. After stirring at room temperature for 3h, the solution was extracted by diethyl ether (150 mL×2). Then the combined organic layers were washed with saturated $NaHCO_3$ solution (75mL×2) and saturated NaS_2O_3 solution (75 mL×2), respectively. Next, the solution was dried with anhydrous sodium sulfate. The filtrate was concentrated in the rotary evaporator and dissolved in solvent (300 mL) with equal volume of n-hexane and t-BuOMe. Finally, the organic layer was

isolated in SAFE apparatus and concentrated to 2 mL by using a rotary evaporator.

The mass spectral data (DB-WAX) and retention indices of the synthesized substances were as follows:

(*Z*)-1-propenyl propyl trisulfide (CAS 23838-26-8): mass spectrometry-electron ionization (MS-EI) m/z (%):180 (100), 41(87), 74 (84), 45(77), 106(47), 73(45), 115(38), 83(34), 39(33), 116(31). RI: 1797 (DB-WAX), 1336 (HP-5MS).

(*E*)-1-propenyl propyl trisulfide (CAS 23838-27-9): MS-EI m/z (%):180 (100), 41(74), 74 (73), 45(65), 106(47), 73(44), 115(38), 83(35), 116(31), 39(27). RI: 1818 (DB-WAX), 1344 (HP-5MS).

(*Z*)-3,6-diethyl-1,2,4,5-tetrathiane (CAS 934273-79-7): MS-EI m/z (%):73(100), 74(69),45(51), 41(51), 212(30), 147(29), 138(24), 39(20), 64(16), 59(11). RI: 2217 (DB-WAX), 1587 (HP-5MS).

(*E*)-3,6-diethyl-1,2,4,5-tetrathiane (CAS 934273-77-5): MS-EI m/z (%):73(100), 74(64), 45(49), 41(26), 147(47),212(29), 138(23), 39(18), 64(14), 58(11). RI: 2235 (DB-WAX), 1592 (HP-5MS).
